# Efficacy of Ultrasound-Guided Injection of Botulinum Toxin, Ozone, and Lidocaine in Piriformis Syndrome

**DOI:** 10.3390/healthcare11010095

**Published:** 2022-12-28

**Authors:** Ahmed Gamal Salah Elsawy, Abdulnasir Hussin Ameer, Yasser A. Gazar, Abdallah El-Sayed Allam, Shun-Ming Chan, Se-Yi Chen, Jin-De Hou, Yu-Ting Tai, Jui-An Lin, Felice Galluccio, Doaa Waseem Nada, Ahmed Esmat

**Affiliations:** 1 Anesthesia and Intensive Care Department, Faculty of Medicine, Al-Azhar University, Cairo 11884, Egypt; 2 Clinical Neurophysiology, Department of Physiology, College of Medicine, Baghdad University, Baghdad 61224, Iraq; 3 Rheumatology and Rehabilitation Department, Faculty of Medicine, Al-Azhar University, Cairo 11884, Egypt; 4 Department of Physical Medicine, Rheumatology and Rehabilitation, Faculty of Medicine, Tanta University, Tanta 31527, Egypt; 5 MoMaRC Morphological Madrid Research Center, Ultra Dissection Group, 28029 Madrid, Spain; 6 Interventional Clinical Neurophysiology Fellowship, Baghdad, Ministry of Health, Baghdad 61224, Iraq; 7 Department of Anesthesiology, Tri-Service General Hospital, National Defense Medical Center, Taipei 11490, Taiwan; 8 Department of Neurosurgery, Chung-Shan Medical University Hospital, Taichung 40201, Taiwan; 9 School of Medicine, Chung-Shan Medical University, Taichung 40201, Taiwan; 10 Division of Anesthesiology, Hualien Armed Forces General Hospital, Hualien 97144, Taiwan; 11 Department of Anesthesiology, School of Medicine, National Defense Medical Center, Taipei 11490, Taiwan; 12 Center for Regional Anesthesia and Pain Medicine, Wan Fang Hospital, Taipei Medical University, Taipei 116, Taiwan; 13 Department of Anesthesiology, Wan Fang Hospital, Taipei Medical University, Taipei 116, Taiwan; 14 Department of Anesthesiology, School of Medicine, College of Medicine, Taipei Medical University, Taipei 116, Taiwan; 15 Center for Regional Anesthesia and Pain Medicine, Chung Shan Medical University Hospital, Taichung 40201, Taiwan; 16 Department of Anesthesiology, School of Medicine, Chung Shan Medical University, Taichung 40201, Taiwan; 17 Department of Anesthesiology, Chung Shan Medical University Hospital, Taichung 40201, Taiwan; 18 Rheumatology & Rehabilitation, Fisiotech Lab Studio, 50136 Firenze, Italy; 19 Neurology Department, Faculty of Medicine, Al-Azhar University, Cairo 11884, Egypt

**Keywords:** botulinum toxins, injections, intramuscular, lidocaine, ozone, piriformis muscle syndrome, ultrasonography, interventional

## Abstract

**Background**: Piriformis syndrome (PS) is a painful musculoskeletal condition characterized by a deep gluteal pain that may radiate to the posterior thigh and leg. This study was designed to compare the effectiveness of ozone and BTX to lidocaine injection in treating piriformis syndrome that was resistant to medication and/or physical therapy. **Study design**: Between November 2018 and August 2019, we involved eighty-four subjects diagnosed with piriformis syndrome in a double-blinded, prospective, randomized comparative study to receive an ultrasound-guided injection of lidocaine (control group), botulinum toxin A, or local ozone (28 patients each group) in the belly of the piriformis muscle. Pain condition evaluated by the visual analog score (VAS) was used as a primary outcome, and the Oswestry Disability Index (ODI) as a secondary outcome, before, at one month, two months, three months, and six months following the injection. **Results**: The majority (58.3%) of patients were male, while (41.7%) were female. At one month, a highly significant decrease occurred in VAS and ODI in the lidocaine and ozone groups compared to the botulinum toxin group (*p* < 0.001). At six months, there was a highly significant decrease in VAS and ODI in the botulinum toxin group compared to the lidocaine and ozone groups (*p* < 0.001). **Conclusion**: Botulinum toxin may assist in the medium- and long-term management of piriformis syndrome, while lidocaine injection and ozone therapy may help short-term treatment in patients not responding to conservative treatment and physiotherapy.

## 1. Introduction

A lesser-known cause of extra-spinal sciatica is piriformis syndrome (PS), in which a number of conditions affecting the piriformis muscle or its vicinity may irritate the nearby sciatic nerve and cause unilateral deep gluteal pain radiating to the ipsilateral thigh. This pain may be worsened by actions that increase piriformis muscle tension, such as rotation of the hip in flexion or knee extension, in addition to sitting intolerance, tenderness over the sciatic notch, and limitation of straight leg raising. These signs are consistent with morphological changes in the piriformis muscle and sciatic nerve [1,2]. Piriformis syndrome has two different varieties. The piriformis muscle or sciatic nerve being divided, or even an abnormal sciatic nerve pathway, are examples of anatomical causes of the primary type, and less than 15% of instances involve it. The secondary type is caused by local ischemia, mass effect, and gluteal macro or microtrauma in the sacroiliac or gluteal regions [3]. Although PS is still a contentious diagnosis for sciatica, routine image studies and electromyography are advised to rule out any potential hip joint or spine issues [4].

With a wide range of therapeutic alternatives, conservative treatment for PS significantly reduces pain and enhances function. Non-steroidal anti-inflammatory drugs, muscle relaxants, and neuropathic pain medications are among the pharmacological treatments included, along with physiotherapy, psychotherapy, lifestyle changes, and local injection procedures [4]. The piriformis muscle is best injected utilizing an ultrasound-guided approach due to its deep placement, small size, and relationship to nearby neurovascular systems [3,4].

Ozone is a soluble allotropic form of oxygen produced by a direct exposure of oxygen to the electric current in ozone generators, and it has excellent oxidizing activity in biological tissues producing reactive oxygen species and lipid oxidation products. These molecules function as biochemical regulators of inflammation through the downregulation of the tumor necrosis factor, as well as tumor necrosis factor receptor 2 [5]. Ozone therapy also has an analgesic effect, which raises the pain threshold by activating serotonin-mediated pathways to release endogenous opioids [6]. Due to the absence of adverse effects or major complications, many studies support its use in treating myofascial pain syndromes and piriformis syndrome [7]. 

Furthermore, due to its distinct mechanism of preventing the release of acetylcholine at the muscle plaque level, botulinum toxin (BTX) has been widely used in numerous sectors of medicine to treat focal hypertonia, muscle spasticity, dystonia, and other diseases [8,9]. The control of neuropeptides, such as substance P, the calcitonin gene-related peptide, glutamate, and the suppression of vanilloid receptor activity, are responsible for the analgesic effect [10,11,12,13]. According to the United States Food and Drug Administration, only two forms of botulinum toxin (serotypes A and B) are permitted for medical usage with beneficial outcomes and fewer adverse effects in neurologic disorders [14].

However, piriformis syndrome frequently develops into a chronic condition; thus, pharmaceutical treatment is only advised for a short time [15]. In order to avoid resorting to invasive operations such as endoscopic decompression of the sciatic nerve with or without the release of the piriformis muscle if conservative treatment fails, additional therapeutic options such as botulinum toxin, ozone injections, and neurological therapy are needed. This study aimed to compare the effectiveness of ozone and BTX to lidocaine injection in treating piriformis syndrome that was resistant to medication and/or physical therapy.

## 2. Patients and Methods

This prospective comparative study was conducted between November 2018 and August 2019 at Al-Azhar University Hospitals (Al-Hussein and Sayed Galal) after local and institutional ethics committee approval. The study included 84 patients, all of whom gave their written informed consent to participate. The research followed the rules of the Declaration of Helsinki regarding human research. We included patients diagnosed with primary piriformis syndrome by history, physical examination, external gluteal tenderness near the sciatic notch, and clinical tests: Freiberg, Pace, and Fair [16,17,18]. The participants were chosen based on their ASA I-III status, age (20–65 yrs), failure of conservative therapy, and visual analog score (VAS) of more than 5 in the morning for six months. When alternative causes of sciatica could not be ruled out, an MRI or X-ray of the hips and lumbar spine was prescribed. Exclusion criteria included ASA IV, BMI ≥ 40, a history of intramuscular injection of corticosteroid within the past three months, non-steroidal anti-inflammatory drugs at least one week before the intervention, a history of allergy to any of the study drugs, neurological or psychiatric disease, local or systemic infection, and previous lumbosacral surgery. The patients were assigned randomly by closed opaque envelopes into the lidocaine group (28 patients) (LD group), the ozone group (28 patients) (OZ group), and the botulinum toxin group (28 patients) (BTX group). They were instructed about the procedure, the drugs used, and possible beneficial or side effects.

### 2.1. Injection Technique

**Figure 1:**
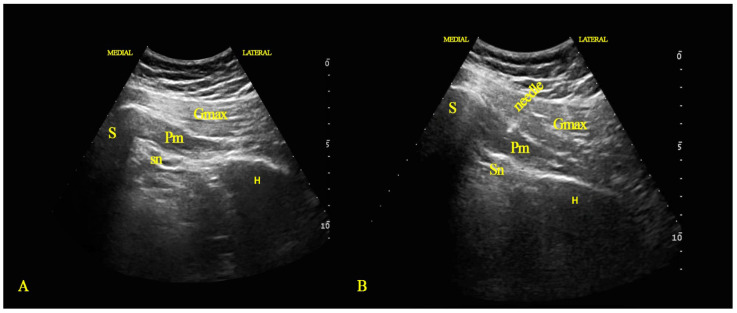
Ultrasound scan (**A**) and ultrasound-guided injection (**B**) for piriformis syndrome. S: sacrum; H: hip bone; Gmax: gluteus maximus muscle; Pm: piriformis muscle; Sn: sciatic nerve.

Each patient was placed in a prone position. The buttock region was scanned with either a 6–13 MHz linear array transducer or a 2–5 MHz curvilinear array transducer (M-Turbo^®^, FUJIFILM Sonosite, Bothell, DC, USA), according to the patient’s build. The transducer was parallel to the piriformis muscle (PM), with the medial side directed to the sacrum and the lateral side towards the great trochanter (Figure 1A). 

The acoustic shadows of the sacrum and the hip bone were used as a reference for the ultrasound visualization of the PM, which is located deeply at the lateral sacral margin as a hypoechoic band between the gluteal muscles and the hip bone. The sciatic nerve appears as an oval honeycombed structure with mixed echogenicity deep in the PM.

The intervention area (i.e., the area of greatest soreness) was sterilized with chlorhexidine and draped. After local infiltration anesthesia, a 22-gauge needle (Stimuplex^®^ Ultra 360^®^, B. Braun Medical Inc., Bethlehem, PA, USA) was advanced under direct ultrasound guidance in an in-plane orientation, traversing the skin, subcutaneous fat, and gluteus maximus, and passing from lateral to medial through the piriformis sheath and into the muscle belly itself (Figure 1B). The ultrasound provided reasonable control to avoid needle penetration into the sciatic nerve or the pelvis. The interventionist injected either 100 U of botulinum toxin A (BOTOX^®^, Allergan, Ireland) diluted to 3 mL, with 0.9% sodium chloride, a mixture of 3 mL of oxygen-ozone (O2-O3) with an ozone concentration of 20 μg/mL (Ozonobaric P^®^ machine; Sedecal, Spain.), or 3 mL of 1% lidocaine (Xylocaine^®^ Lidocaine Hydrochloride Injection USP, Astra Zeneca), according to the group. All patients were evaluated for pain intensity and disability before the intervention, and then at one month, two months, three months, and six months after the intervention.

The primary outcome measure included the average pain intensity assessed using VAS from 0 to 10, where 0 = no pain and 10 = worst possible pain. The secondary outcome measure included the Oswestry Disability Index (ODI), which is a self and subjective expression of the extent of disability and is composed of ten questions, each with six choices. The questions cover the pain intensity; ability to work, stand, sit, lift, and perform personal care; travel; sleep pattern; and sexual life. A score of 0–5 is given to each question to sum it and present the sum as a percentage of 100 [18].

### 2.2. Statistical Analysis

Regarding sample size calculation, we planned a study of a continuous response variable from independent control and interventional subjects with one control(s) per interventional subject. In a previous study [19], each subject group’s response was normally distributed with a standard deviation of 0.2. Suppose the actual difference between study and control means is 0.2; twenty-eight subjects each for the study and control groups were needed to reject the null hypothesis that the population means of the study and control groups are equal with a probability (power) of 0.8. The type I error probability associated with this null hypothesis test is 0.05. Data entry and statistical analysis were carried out using MedCalc ver. 18.11.3 (MedCalc, Ostend, Belgium). Tests of significance were used, including the Chi-square test, analysis of variance (ANOVA), and post hoc Tukey’s tests. 

## 3. Results

All patients completed the study. The average age of all patients was 39.5 ± 8.58 years, and the average body mass index was 29.5 ± 2.9 kg m^−2^. 

Most patients were male (58.3% male, 41.7% female), with a mean symptoms’ duration of 10.9 ± 2.5 months, a VAS score of 8 ± 0.92, and an ODI score of 45.6 ± 9.9. The demographic data in each group are shown in (Table 1).

No significant statistical difference was seen between the three groups for all demographic data. (Table 1). Regarding pre-injection VAS, our results revealed a non-statistical difference between the three studied groups. However, ODI was significantly increased in the BTX as well as the OZ group (0.003, 0.053, respectively) (Table 2).

Overall, the values of VAS and ODI decreased after one month (VAS 3.88 ± 2.97; ODI 34.36 ± 10) and then remained almost stable, with a general trend of slight progressive increase in the second month (VAS 3.9 ± 1.9; ODI 31.6 ± 3.8), third month (VAS 4.1 ± 1.2; ODI 32.8 ± 4.5), and sixth month after injection (VAS 4.6 ± 2.4; ODI 34.2 ± 9.9). The comparative analysis between the three groups showed a highly significant decrease in the first and second month, in both VAS and ODI, in the LD and OZ groups, compared to the BTX group, with the inter-group differences demonstrated in Table 3. 

**Figure 2:**
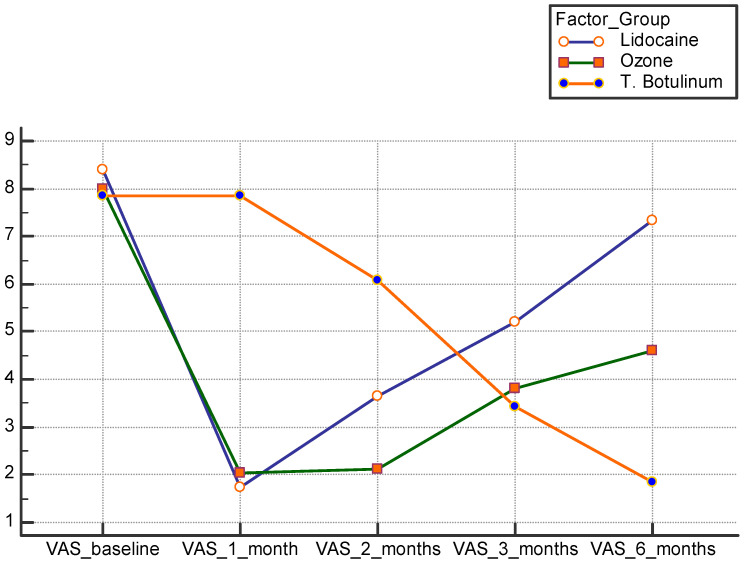
Post-injection comparison between the three studied groups as regards VAS score. VAS: visual analog scale. T. botulinum: botulinum toxin.

**Figure 3:**
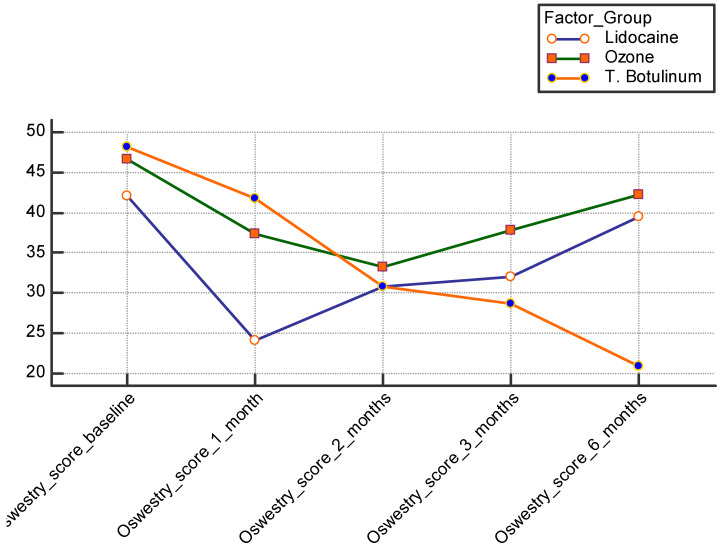
Post-injection comparison between the three studied groups as regards ODI score. ODI: Oswestry Disability Index.

After two months, both VAS and ODI scores remained almost stable for the OZ group, while we noted a progressive increase in the LD group; the reduction in both scores started to occur in the BTX group after two months (Figure 2 and Figure 3). From the third month post injection, the values of VAS and ODI significantly decreased only in the BTX group compared to the LD and OZ groups (Figure 2 and Figure 3).

### Adverse Events

Nearly all patients in the three study groups experienced success with the US-guided technique, and no problems or adverse effects were noticed.

## 4. Discussion

This study shows promising options for chronic cases of PS after the failure of conservative therapy. By injecting lidocaine, ozone, or BTX, we can significantly improve both short- and long-term pain and disability scores. Nearly all patients involved in the three study groups experienced success with the US-guided injection technique, with no adverse events noticed.

A significant portion of PS cases remain undiagnosed even though the incidence ranges from 5% to 36% [20,21,22]. This issue stems from the lack of standardized diagnostic criteria for diagnosing PS, and there are currently continuing discussions regarding the syndrome’s advantageous diagnostic and treatment choices, given the many theories explaining its etiopathogenesis. When PS is clinically suspected, local anesthetic injection into the belly of the PM can result in a 50% reduction in the patient’s symptoms, which is why many authors regard this treatment as a crucial diagnostic tool [3,23,24]. However, some recent material supports the use of diagnostic scores [25] and electromyographic signals in the diagnosis [26]. 

Injections into the peri-sciatic nerve, the medial aspect of the muscle, or the side, are just a few of the different injection strategies discussed in the treatment of PS [27]. However, no clear tests have been carried out to determine which strategy is best. In our study, we found that it was preferable to administer the injections directly into the area of greatest soreness while using ultrasound guidance to advance through the piriformis muscle sheath and into the actual muscle belly.

The recruited patients’ average age was 39.5 ± 8.58 years, and the average BMI was 29.5 ± 2.9 kg m^−2^. Regarding the gender of the studied patients, the majority (58.3%) were male, while 41.7% were female. As the female: male ratio in PS was reported to be 6:1, these results did not accord with those previously recommended in the literature. Additionally, Shah et al.’s [1] research showed that primary PS is more common in young females than older males. 

After 1 and 2 months of follow-up, our study showed that VAS and Oswestry Disability scores in the lidocaine and ozone groups significantly decreased compared to the BTX group (*p* < 0.001), which concurred with the finding of Kongsagul et al. [22] that local anesthetics are as effective as corticosteroids when administered intramuscularly or in the interfacial plane containing nerve terminals. Local anesthetics have anti-inflammatory effects via directly altering polymorphonuclear leukocytes as well as macrophage and monocyte activity, in addition to preventing the peripheral and central sensitization that results from myofascial trigger points via the generated reactive hyperemia [22,28,29].

Additionally, Valdenassi et al. [8] described a reduction in VAS after intramuscular ozone injection, from 9.1 to 5.6 at mid-treatment (after two weeks) and 2.2 at the end (after four weeks), to 0.4 at follow-up (eight weeks of injection). However, Paoloni and his colleagues [30] demonstrated that the ozone effect may continue for six months after intramuscular injection in patients with low back pain, and sixty-one percent of patients became pain-free six months after treatment. Moreover, Melchionda et al. [31] exhibited an 80% success rate for oxygen-ozone treatment at the six-month follow-up versus 50% for the non-steroidal anti-inflammatory drugs.

Ozone not only stimulates the central nervous and endocrine systems, but also improves hormonal production, neurotransmitter release, and metabolism, leading to an increase in pain threshold [32,33]. Ozone therapy also increases the production of lipid oxidation products and endogenous antioxidants; moreover, it raises the serum level of interleukin 8, which promotes the phagocytosis of bacteria and necrotic tissue and aids tissue healing [6]. Furthermore, ozone inhibits the production of inflammatory prostaglandins by modifying the breakdown of arachidonic acid and kick-starts the healing process by activating fibroblasts [34].

The results from our comparative study among the three groups at 3 and 6 months also showed a highly significant decrease in VAS and Oswestry Disability scores in the BTX group compared with the lidocaine and ozone groups (*p* < 0.001). This result is supported by a placebo-controlled trial on 145 myofascial pain patients, which revealed that BTX is valuable for controlling pain severity and duration in myofascial pain syndrome for 4–6 weeks after the injection [35]. Moreover, Santamato A and colleagues found that treatment with abo-botulinum toxin A for PS produced a high therapeutic effect that is superior to steroids and placebo. A higher percentage (65%) of patients who received injections of abo-botulinum toxin A experienced a 50% reduction in VAS score than patients who received lidocaine with steroids (32%) [36]. Moreover, the effectiveness of BoNT-A injection into the piriformis muscle was demonstrated by Michel and his colleagues in 122 participants who had previously undergone pain management procedures without experiencing any pain relief [25]. They injected a dosage of botulinum toxin A ranging from 50 to 100 U under the supervision of an EMG. Three months was the minimum between injections. The VAS was used to assess the injected patients: in 77% of the cases, pain relief was rated as “very good”, “average” in 7.4%, and “bad” in 15.6% of the cases. Additionally, the effects of BTX are claimed to peak one to four weeks after injection and subside three to four months later, according to cosmetic practice [37].

In contrast, some studies claimed that BTX did not improve the VAS (20% at 15 days and 30 days, and 22% at 90 days after treatment, *P* < 0.05) or the physical performance when compared to sodium chloride at 0.9% or bupivacaine at 0.25% in 27 patients complaining of low back pain [38]. The same conclusion was disclosed by Qerama et al. [39] and Ojala et al. [40] in patients with chronic myofascial pain. Minghe et al. [41] also reported that, currently, there are insufficient data to quantify pain reduction in PS patients treated with BTX injection. This may be explained by the hypothesis that BTX is absorbed only by pinocytotic pathways and is, therefore, not very effective, as well as that many nerve cells lack specialized BTX receptors [42]. Additionally, the inflammatory response might have decreased the tissue concentration of BTX, which would have led to diminished efficacy [43]. Our findings were based on the fact that, in order to maintain pain relief and for long-term recovery, BTX weakens muscles that are in acute pain and breaks the cycle of repeated muscle spasms and pain in PS patients. Although the full nature of BTX’s painkilling mechanism of action is still unknown, it may involve multiple factors [36]. It has been established that BTX inhibits the release of a variety of chemical mediators, such as lactate, potassium H+ ions, bradykinins, serotonin, prostaglandin E2, and neuropeptides such as substance P and calcitonin gene-related peptide, which increase muscle pain in PS through central and peripheral sensitization. These mediators are produced as a result of a muscle’s protracted contraction [11,13]. Acetylcholine secretion is thought to correlate with muscular spasm, although there is still some disagreement. It has been experimentally demonstrated that BTX suppresses other neurotransmitters such as norepinephrine in the same mode of action as it destroys SNARE complexes, despite the fact that BTX’s specificity on cholinergic nerves is owing to unique receptors in the nerve terminal membrane. As it also inhibits the release of other neurotransmitters and neuropeptides, BTX has powerful anti-acetyl cholinergic and anti-inflammatory properties [36].

Thus came the significance of ultrasound-guided BTX or ozone injections, as preferred to electromyography, fluoroscopy, CT, or MRI-guided injection procedures, which have been utilized to increase the accuracy of needle placement into the PM for the treatment of chronic resistant cases of PS. However, it was shown that these methods were not adequate for determining the needle depth required to reach the PM [44]. Additionally, Smith et al. [5] stated that ultrasound-guided injections have many benefits and are superior to EMG, fluoroscopy, CT, and MRI, because they are easy, affordable, and readily available. It was noted that, in comparison to the procedures previously mentioned, ultrasound has no known contraindications, emits no ionizing radiation, is well tolerated by patients, and does not call for contrast material. Ultrasound offers real-time viewing of the needle’s progress towards an intended target, in addition to exceptional soft tissue resolution, bone landmark identification, and nerve and vessel identification [44,45,46].

## 5. Conclusions

While lidocaine and ozone therapies may aid short-term treatment in patients who are not responsive to conservative treatment and physical therapy, BTX may help with the medium- and long-term management for PS patients. The precise mechanism of BTX and ozone therapy on various chemical and inflammatory mediators, as well as their impact on reducing pain and functional disability in chronic resistant PS cases, requires further study with a large sample size.

## Figures and Tables

**Table 1:** Demographic data of the three studied groups.

Variable		LD Group (28)	OZ Group (28)	BTX Group (28)	ANOVA Test
		Mean ± SD	Mean ± SD	Mean ± SD	*p*-Value
Age (years)		37 ± 8.6	41.3 ± 8.3	40.2 ± 8.5	0.149
BMI (kg/m^2^)		29.5 ± 2.7	29.9 ± 3.3	29.1 ± 2.7	0.593
Duration (months)		10.3 ± 2	10.57 ± 2.1	11.8 ± 3.1	0.064
					Chi-Square Test *p*-Value
Gender	Female	10 (35.7%)	14 (50%)	11 (39.3%)	0.529
	Male	18 (64.3%)	14 (50%)	17 (60.7%)	

ANOVA: analysis of variance, BMI: body mass index.

**Table 2:** Baseline (pre-injection) data of the three studied groups.

	LD Group (28)	OZ Group (28)	BTX Group (28)	ANOVA Test	Post Hoc Tukey’s Test
				*p*-Value	*p*-Value
VAS Score	8.4 ± 0.83	8 ± 1	7.8 ± 0.8	0.079	P1 0.291 P2 0.082 P3 0.174
ODI (%)	42 ± 8.6	46.6 ± 9.97	48.2 ± 10.3	0.033 *	P1 0.053 * P2 0.003 * P3 0.056 *

ANOVA: analysis of variance. ODI: Oswestry Disability Index. * significant *p* value ≤ 0.05 VAS: visual analog scale. P1 = comparison between LD group and OZ group, P2 = comparison between LD group and BTX group, P3 = comparison between OZ group and BTX group, Tukey’s test: to analyze VAS and ODI data among the three studied groups before injection.

**Table 3:** Outcome measurement (1-, 2-, 3-, and 6-months post-injection) of the three studied groups.

		LD Group (28)	OZ Group (28)	BTX Group (28)	ANOVA Test	Post Hoc Tukey’s Test
		Mean ± SD	Mean ± SD	Mean ± SD	*p*-Value	*p*-Value
1 month	VAS score	1.75 ± 0.75	2 ± 1.1	7.8 ± 0.9	<0.001 **	P1 0.127 P2 0.001 * P3 0.002 *
	ODI	24 ± 4.4	37.3 ± 7.4	41.7 ± 7.6	<0.001 **	P1 0.035 * P2 0.003 * P3 0.043 *
2 months	VAS score	3.6 ± 1.3	2.1 ± 0.9	6 ± 0.85	<0.001 **	P1 0.073 P2 0.041 * P3 0.024 *
	ODI	30.8 ± 2.88	33.3 ± 5.4	30.8 ± 2.1	0.019 *	P1 0.041 * P2 0.083 P3 0.068
3 months	VAS score	5.2 ± 1.2	3.8 ± 0.9	3.4 ± 0.9	<0.001 **	P1 0.056 * P2 0.054 * P3 0.081
	ODI	32 ± 8.3	37.8 ± 3.1	28.6 ± 1.8	<0.001 **	P1 0.70 P2 0.0581 * P3 0.001 *
6 months	VAS score	7.3 ± 0.77	4.6 ± 1	1.8 ± 0.7	<0.001 **	P1 0.040 * P2 0.001 * P3 0.004 *
	ODI	39.5 ± 3.5	42.2 ± 2.5	20.9 ± 2.7	<0.001 **	P1 0.70 P2 0.055 * P3 < 0.009 *

VAS: visual analog scale, ODI: Oswestry Disability Index. P1 = comparison between LD group and OZ group, P2 = comparison between LD group and BTX group, P3 = comparison between OZ group and BTX group. Tukey’s test was used to analyze VAS and ODI data among the three studied groups in each post-injection period. * significant *p* value ≤ 0.05, ** highly significant *p* value ≤ 0.001.

## Data Availability

We provide the data in Supplementary Materials.

## Supplementary Materials

The following supporting information can be downloaded at: https://www.mdpi.com/article/10.3390/healthcare11010095/s1.
