# Supplementary material for: Efficacy of Ultrasound-Guided Injection of Botulinum Toxin, Ozone, and Lidocaine in Piriformis Syndrome
Source: Healthcare (Basel). 2022 Dec 28;11(1):95. doi: 10.3390/healthcare11010095 (PMC9818865; doi:10.3390/healthcare11010095)
Supplement: Supplementary file 1 [file healthcare-11-00095-s001.zip › healthcare-2067904-supplementary.pdf]

Number	Name	Group	Baseline (pre-injection) data						Outcomes (1-month)		Outcomes (2-months)		Outcomes (3-months)		Outcomes (6-months)	
			Age (years)	Sex ^^Male = M ^^Female = F	BMI	Duration of symptoms (month)	VAS_baseline	Oswestry score_baseline%	VAS_1-month	Oswestry score_1-month	VAS_2-months	Oswestry score_2-months	VAS_3-months	Oswestry score_3-months	VAS_6-months	Oswestry score_6-months
1	236417	Lidocaine	38	M	31	8	8	35	2	24	4	33	5	32	7	34
2	215404	Lidocaine	29	F	29	13	7	42	1	26	3	35	4	34	6	40
3	218463	Lidocaine	40	M	33	9	8	49	3	20	3	31	6	32	8	38
4	117806	Lidocaine	54	F	27.5	7	9	53	2	29	4	29	5	29	7	45
5	276073	Lidocaine	32	M	28	14	7	31	1	18	2	30	5	31	8	40
6	169495	Lidocaine	31	M	29	9	9	33	1	23	5	32	4	31	8	33
7	151144	Lidocaine	30	M	30	12	8	41	1	25	4	36	5	35	7	41
8	260737	Lidocaine	41	M	32	10	9	50	3	21	4	32	7	33	8	37
9	244770	Lidocaine	55	F	27	8	9	52	2	30	5	30	5	28	8	45
10	66864	Lidocaine	33	M	28	14	8	28	1	20	2	29	6	32	7	40
11	280701	Lidocaine	35	M	31	10	10	39	2	26	4	33	7	34	8	36
12	274779	Lidocaine	29	F	27	11	8	40	1	20	2	35	4	31	6	40
13	237717	Lidocaine	42	M	36	11	9	54	3	29	5	29	6	31	7	41
14	144187	Lidocaine	51	F	25	7	7	49	2	29	1	30	3	30	7	43
15	207701	Lidocaine	31	M	30	12	9	31	2	20	5	26	7	30	8	39
16	281508	Lidocaine	26	F	30	11	7	35	1	20	2	29	3	31	7	34
17	265229	Lidocaine	32	M	31	10	9	41	2	21	6	31	5	37	9	41
18	272230	Lidocaine	41	M	32	9	9	52	3	32	6	34	7	36	7	39
19	265575	Lidocaine	35	M	30	9	9	38	1	25	3	32	6	33	7	35
20	257829	Lidocaine	30	F	28	12	9	41	1	21	3	36	5	32	7	41
21	277375	Lidocaine	41	M	35	10	9	53	2	28	4	28	5	30	6	40
22	241634	Lidocaine	52	F	26	8	8	50	3	30	2	31	4	31	8	44
23	272135	Lidocaine	30	M	29	11	8	30	1	19	4	25	6	29	7	38
24	173017	Lidocaine	27	F	31	12	7	36	2	21	3	30	4	32	7	35
25	265342	Lidocaine	31	M	30	9	9	40	1	20	5	30	4	36	8	40
26	65885	Lidocaine	40	M	31	8	8	51	2	31	5	33	6	35	6	38
27	274293	Lidocaine	50	F	25	11	9	54	1	27	4	29	5	33	8	46
28	272038	Lidocaine	31	M	26	13	9	30	2	18	2	26	7	29	8	44
		mean	37.04		29.55	10.29	8.39	42.07	1.75	24.04	3.64	30.86	5.21	32.04	7.32	39.54
		SD	8.63	#DIV/0!	2.71	2.00	0.83	8.64	0.75	4.41	1.34	2.89	1.20	2.33	0.77	3.52
29	193183	Ozone	28	F	34	10	6	59	1	46	1	40	3	42	3	42
30	278283	Ozone	47	M	30	12	8	48	2	37	2	35	4	35	4	43
31	260018	Ozone	51	F	26	13	9	55	2	43	3	35	3	39	5	44
34	257730	Ozone	36	M	33.5	7	7	39	1	33	2	29	5	38	6	45
35	282521	Ozone	44	F	27	9	6	33	3	25	2	25	4	34	5	36
36	266123	Ozone	29	F	35	11	7	60	2	47	2	41	4	43	4	43
37	287837	Ozone	46	M	29	12	9	49	3	38	3	36	5	36	5	44
38	287837	Ozone	50	M	25	12	8	54	1	42	2	34	2	38	4	43
39	213214	Ozone	37	M	32	7	8	38	1	32	1	28	4	37	5	44
40	226337	Ozone	45	F	28	10	7	34	3	26	2	26	4	35	5	37
41	220615	Ozone	30	F	36	12	8	31	3	48	3	42	5	44	5	44
42	73834	Ozone	47	M	30	12	8	48	2	37	2	35	4	35	4	43
43	281789	Ozone	51	F	26	13	9	55	2	43	3	35	3	39	5	44
44	244721	Ozone	34	M	31	6	5	37	1	31	1	27	3	36	4	43
45	211417	Ozone	46	F	29	11	8	35	5	27	4	27	6	36	7	38
46	278122	Ozone	29	F	35	11	7	60	2	47	2	41	4	43	4	43
47	210471	Ozone	44	M	27	10	8	47	1	36	1	33	3	34	3	41
48	271472	Ozone	51	M	26	13	9	55	1	40	2	35	3	39	5	44
49	282909	Ozone	29	F	35	11	7	60	2	47	2	41	4	43	4	43
50	283414	Ozone	46	M	29	11	7	47	1	36	1	34	3	34	3	42
51	213534	Ozone	52	F	27	14	10	56	3	44	4	36	4	40	6	45
52	251109	Ozone	35	M	32	7	6	38	1	32	1	28	4	37	5	44
53	188971	Ozone	45	F	28	10	7	34	4	26	3	26	5	35	6	37
54	163663	Ozone	28	F	34	10	6	59	1	46	1	40	3	42	3	42
55	114280	Ozone	45	M	28	11	9	48	2	37	2	35	4	35	4	42
56	181568	Ozone	51	M	26	13	9	55	2	41	3	35	3	39	5	44
57	97614	Ozone	36	M	30	7	7	37	1	31	1	27	3	36	4	43
58	260816	Ozone	46	F	29	11	8	35	4	27	3	27	5	36	6	38
		mean	41.36		29.91	10.57	7.61	46.64	2.04	37.32	2.11	33.32	3.82	37.86	4.61	42.18
		SD	8.34		3.31	2.13	1.20	9.98	1.10	7.40	0.92	5.40	0.90	3.14	1.03	2.55
59	274166	T. Botulinum	54	M	29	14	9	42	9	40	6	30	3	26	1	22
60	218884	T. Botulinum	39	F	33	10	8	49	8	37	7	33	4	29	3	18
61	276134	T. Botulinum	38	M	28	16	6	61	6	50	5	28	3	27	2	24
64	179181	T. Botulinum	41	F	25.5	11	8	33	8	32	6	35	3	30	3	19
65	210373	T. Botulinum	27	M	30.5	7	7	55	7	49	5	27	4	31	1	21
66	93206	T. Botulinum	55	M	30	15	9	43	9	41	7	31	4	27	2	23
67	285395	T. Botulinum	38	F	32	9	7	48	7	36	6	32	3	28	2	17
68	128844	T. Botulinum	39	M	29	17	7	62	7	51	6	29	4	28	3	25
69	271469	T. Botulinum	40	F	24	10	7	32	7	31	5	34	2	29	2	18
70	139688	T. Botulinum	28	M	31	8	8	54	8	50	6	28	4	30	2	22
71	119163	T. Botulinum	54	M	29	14	9	42	9	40	7	30	3	26	1	22
72	263622	T. Botulinum	39	F	31	10	8	49	8	37	7	33	4	29	3	18
73	146784	T. Botulinum	38	M	28	16	7	61	6	50	5	28	3	27	2	24
74	245493	T. Botulinum	39	F	24	9	9	31	7	30	4	33	2	28	1	17
75	236808	T. Botulinum	29	M	32	9	8	57	8	51	7	29	6	33	2	23
76	242949	T. Botulinum	53	M	30	13	9	40	9	41	7	31	4	26	1	22
77	244010	T. Botulinum	40	F	31	10	8	51	8	36	7	30	3	29	1	18
78	184120	T. Botulinum	36	M	27	15	7	60	8	50	6	31	3	30	2	24
79	260018	T. Botulinum	55	M	30	15	9	43	9	41	7	31	4	27	2	23
80	143301	T. Botulinum	38	F	32	9	7	48	7	36	6	32	3	28	2	17
81	294008	T. Botulinum	39	M	29	17	7	62	7	51	6	29	4	28	3	25
82	118484	T. Botulinum	40	F	25	10	8	32	8	31	5	34	2	29	2	18
83	199816	T. Botulinum	28	M	31	8	8	56	8	50	6	28	5	32	1	22
84	293218	T. Botulinum	54	M	29	14	9	42	9	40	7	30	4	26	1	22
85	274472	T. Botulinum	39	F	33	10	8	50	8	37	7	33	4	29	2	18
86	142676	T. Botulinum	38	M	28	16	7	61	8	50	6	30	3	30	2	24
87	34189	T. Botulinum	39	F	23	10	8	31	9	30	5	33	2	29	1	19
88	269518	T. Botulinum	29	M	31	9	8	54	8	51	6	30	3	31	2	21
		mean	40.21		29.11	11.82	7.86	48.18	7.86	41.75	6.07	30.79	3.43	28.64	1.86	20.93
		SD	8.50		2.76	3.15	0.85	10.29	0.89	7.66	0.86	2.15	0.92	1.83	0.71	2.69
